# Impact of heat stress on embryonic development during first 16 days of gestation in dairy cows

**DOI:** 10.1038/s41598-021-94278-2

**Published:** 2021-07-21

**Authors:** Ramanathan Kasimanickam, Vanmathy Kasimanickam

**Affiliations:** 1 Department of Veterinary Clinical Sciences, College of Veterinary Medicine, Washington State University, Pullman, WA 99164 USA; 2 AARVEE Animal Biotech LLC, Corvallis, OR 97333 USA

**Keywords:** Developmental biology, Climate sciences

## Abstract

Objective was to elucidate the effects of heat stress (HS) on embryo development during first 16 gestational days (GD) and circulating hormone concentrations on GD-16 in lactating Holstein cows. Cows in HS and control (CON) groups were exposed to temperature humidity index (THI) of ≥ 73 and < 73, respectively, for 3 weeks before the experiment. GD-7 (67 vs 49%) and GD-16 (52 vs. 31%) conception rates following single insemination were greater (*P* < 0.01) for CON compared with HS cows. Control cows produced more GD-7 transferrable embryos following superovulation compared with HS cows (84.8 vs 53.1%; *P* < 0.001). Mean (± SEM) length (45.2 ± 10.6 vs. 59.2 ± 9.1 mm) and weight (31.4 ± 4.3 vs. 42.4 ± 6.2 mg) of GD-16 conceptus were greater for CON compared with HS cows (*P* < 0.05). Control cows yielded more filamentous conceptus (≥ 25 mm) compared with HS cows (71 vs 45%; *P* < 0.05). Progesterone (2.09-fold) was higher, and cortisol (1.86-fold), prolactin (1.60-fold), substance-P (1.55-fold), Isoprostane-8 (1.34-fold) and prostaglandin F metabolites (1.97-fold) were lower in CON compared with HS cows (*P* < 0.05). Progesterone positively, and substance-P, isoprostane-8 and the THI negatively were associated with GD-16 conceptus length (*P* < 0.05). In conclusion, altered hormones concentrations in heat-stressed cows plausibly resulted in lower GD-7 and GD-16 conception rates, fewer GD-7 transferable embryos, and stunted GD-16 conceptus elongation.

## Introduction

Heat stress causes an annual economic loss of $900 million to the US dairy industry from decreased milk production, reduced reproductive performance, and increased culling^1^. Heat stress is a major contributing factor to the reduced reproductive performance in dairy cows^2–5^. Several factors trigger the heat stress, but the most imperative elements are increased temperature and humidity that result in poor reproductive performance^2^. Cattle exposed to heat stress show reduced expression of estrus, and experience decreased fertilization rate and increased embryonic mortality^3–8^.

Heat stress during the period of breeding was linked to reduced conception in dairy cows^2^. Furthermore, negative effects of heat stress have been observed from 42 days before to 40 days after breeding^9^. Schüller et al. (2014) investigated the relationship between temperature-humidity index (THI) and conception rate (CR) in lactating dairy cows and found that a THI of ≥ 73 adversely influenced the CR^10^. The authors observed that the mean THI of 73 or more for an hour exposure per day decreased the CR significantly. Further, the drastic negative impact of heat stress was noted from 21 to 1 day before breeding, and the CR decreased from 31 to 12%, correspondingly.

The secretion of glucocorticoids is the classic endocrine response to stress. However, variable endocrine changes occur in response to stress. Within seconds to minutes following stress, increased catecholamines, cortisol releasing hormones (CRH) and adrenocorticotropic hormone (ACTH), decreased gonadotropin-releasing hormone (GnRH), gonadotropins, prolactin, and glucogans secretions occur^11,12^. In addition, over hours to days, gonadal steroid hormone declines^13^. In contrast, Bridges et al. (2005) observed a higher concentration of progesterone in acute response studies associated with adrenal secretion of progesterone or to the severity of the thermal stress^14^. However, a greater drop in progesterone is typically observed when cows are imposed to long-term, chronic, seasonal heat stress^15^.

Heat stress impedes embryonic development^16^ and escalates early embryonic loss. It affects the embryo at its pre-attachment stage, but the magnitude of the impact decreases as the embryo develops^17^. Embryonic survival was significantly reduced by heat stress in pregnant cows during Days 0 to 3 or Days 0 to 7 of pregnancy^18,19^. The effects of heat stress on embryonic survival diminish as pregnancy advances. The viability and development of embryos on Day 8 were hindered by heat stress when superovulated cows were exposed to heat stress on Day 1 but not on Day 3, 5, or 7^20^. The detrimental effect of heat stress to cause embryonic mortality is lessened after the first few days of pregnancy because of the increased resistance of embryos to cellular disruption by the elevated temperature. Several of these documentations are based on the recovery of early embryo subsequent to superovulation or CR at pregnancy diagnosis. Case–control studies on the mechanisms by which heat stress harms early embryo development considering the specific periods, from gestational day (GD) 8 to 16, are lacking. Thus, the objectives of the present study were to elucidate the effects of imposing heat stress (at least for 3 weeks prior) on embryo development between GD-1 and GD-16 in dairy cows. Specifically, the study aims (1) to investigate the effect of heat stress (3 weeks prior to initiation of superovulation treatment) on the embryonic development between GD-1 and GD-7 by means of assessing CR on GD-7 following single insemination and the response to superovulation in dairy cows, and (2) to investigate the effect of heat stress (3 weeks prior to artificial insemination) on the embryonic development between GD-1 and GD-16 by means of assessing embryo morphometry and CR on GD-16. In addition, concentrations of progesterone, cortisol, prolactin, substance-P, Isoprostane-8, and prostaglandin F metabolites (PGFM) were determined to elucidate their association with embryonic development during this period.

## Results

### Experiment 1

#### Experiment 1a

A total of 116 embryos were collected. The CR on GD-7 was greater for CON cows compared with the HS counterpart (63 vs 43%; *P* < 0.01). The embryo quality and quantity for all collections was given in Table 1. The percentage of transferable embryos recovered were greater for the CON cows compared with the HS cows (92.1 vs 60.5%; *P* < 0.01). The percentage of morula was lesser for the CON cows compared with the HS cows (36.2 vs 57.7%; *P* < 0.05). The percentage of blastocysts [63.8 vs 42.3%); *P* < 0.05] were greater, and unfertilized oocytes (4 vs 21%; *P* < 0.01) and degenerate embryos (7.9 vs 39.5%; *P* < 0.01) were lesser for the CON cows compared with the HS cows. In CON cows, 38, 8 and 12 embryos, and in HS cows, 10, 11 and 5 embryos were graded as code 1, code 2 and code 3 embryos, correspondingly.

**Table 1:** Mean (± SEM) number of CL, total embryo recovered, transferrable embryo, morula and blastocyst for dairy cows following single insmeination^1^ under control^2^ and heat stress conditions^3^

Treatment	Cows inseminated	# Total embryo (%)^4^	# UFO (%)^5^	# Transferrable embryos (%)^6^	# Morula (%)^7^	# Blastocyst (%)^7^	# DGM (%)^8^
Control	100	63 (63)	4 (4)	58 (92.1)	21 (36.2)	37 (63.8)	5 (7.9)
Heat stress	100	43 (43)	21 (21)	26 (60.5)	15 (57.7)	11 (42.3)	17 (39.5)

ab, Different superscripts within column were significant (*P* < 0.05);

^1^Refer Fig. 3a for protocol;

^2^Control condition, temperature and humidity index (THI) < 73;

^3^Heat stress condition, THI ≥ 73;

^4^Total embryo (%) = Total embryos recovered /Number inseminated;

^5^UFO (%)—Unfertilized oocytes/Number inseminated;

^6^Transferrable embryos (%) = Number of transferrable embryo/ Number of total embryo;

^7^Morula/Blastocyst (%) = Number of morula/ Number of transferrable embryo (or) number of blastocyst/ Number of transferrable embryo;

^8^DGM (%)—Degenerate embryos/ Number of total embryo.

Rectal temperatures (°C) in the HS and CON groups were 38.1 ± 1.1 and 37.9 ± 1.3 on Day-10, 38.3 ± 1.0 and 37.8 ± 0.9 on Day 0, and 38.6 ± 0.9 and 38.2 ± 1.0 on Day 7, respectively (*P* > 0.1).

#### Experiment 1b

Response to superovulation for cows under heat-stress and control conditions were given in Table 2. Considering the heat stress category, the % total ova and embryos collected on GD-7 were greater for the CON cows compared with the HS cows (85.9% vs 75.3%; *P* < 0.001). The % number of transferrable embryos were greater for the CON cows compared with the HS cows (84.8% vs 53.1%; *P* < 0.001). The % morulas (46.8% vs 31.2%; *P* < 0.01), blastocysts (38.0% vs 21.9%; *P* < 0.01) and UFO/DEM (15.2% vs 46.9%; *P* < 0.01) were greater for the CON group compared with the HS cows. Percentages of superovulation that yielded no embryos for cows in the CON and HS groups were 10% and 20%, correspondingly.

**Table 2:** Mean (± SEM) number of CL, total embryo recovered, transferrable embryo, morula and blastocyst following SO treatment^1^ for dairy cows under control and heat stress conditions^2^

Treatment	# SO	# CL	# SO with zero response^3^	Total ova and embryos (%)^4^	Transferrable embryos (%)^5^	# Morula (%)^6^	# Blastocyst (%)^7^	# UFO/DGM (%)^8^
Control	20	9.2 ± 1.3	2	7.9 ± 1.6 (85.9)^a^	6.7 ± 1.3 (84.8)^a^	3.7 ± 0.6 (46.8)^a^	3.0 ± 0.8 (38.0)^a^	1.2 ± 0.6 (15.2) ^a^
Heat stress	20	8.5 ± 1.0	4	6.4 ± 1.2 (75.3)^b^	3.4 ± 1.6 (53.1)^b^	2.0 ± 0.5 (31.2)^b^	1.4 ± 0.4 (21.9)^b^	3.0 ± 1.3 (46.9) ^b^

ab, Different superscripts within column were significant (*P* < 0.05);

CL—Corpus lutea; SO—Superovulation; UFO—Unfertilized oocytes; DGM—Degenerate embryos;

^1^Refer Fig. 3b for protocol;

^2^Control condition, temperature and humidity index (THI) < 70; Heat stress condition, THI ≥ 73;

^3^Zero response is defined as cows with no CL and zero embryo yield following superovulation treatment.

^4^Total ova and embryos (%) = Total ova and embryos / Number of CL;

^5^Transferrable embryos (%) = Number of transferrable embryo / Number of total ova and embryo;

^6^Morula (%) = Number of morula / Number of total ova and embryo;

^7^Blastocyst (%) = Number of blastocyst / Number of total ova and embryo;

^8^UFO/DFM (%)—UFO/DFM / Number of total ova and embryo.

#### Serum hormone concentrations on gestational Day 7

**Figure 1:**
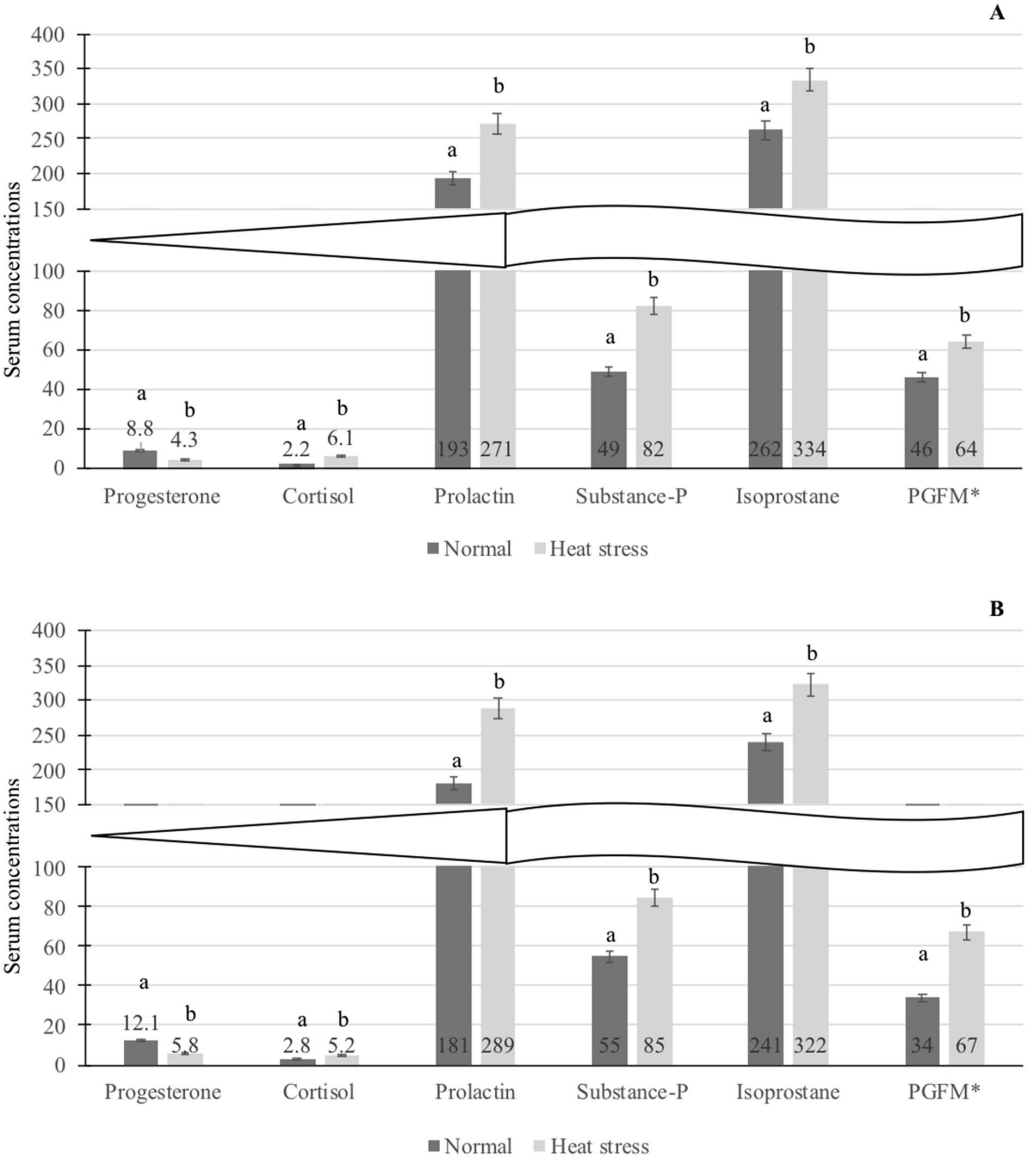
(**A**) Mean (± SEM) serum hormones concentrations on gestational Day 7 (on the day of embryo collection following superovulation) in control and heat stressed dairy cows. (**B**) Mean (± SEM) serum hormones concentrations on gestational Day 16 (following artificial insemination) in control and heat stressed dairy cows. ab, Hormone concentrations with different superscripts differed between control and heat-stressed cows. *PGFM serum concentration is given in pg/mL; Serum progesterone, cortisol, prolactin, substance-P and isoprostane are given in ng/mL.

The mean (± SEM) serum progesterone concentration was greater and cortisol, prolactin, substance-P, Isoprostane-8 and PGFM levels were lesser in cows under control condition compared with cows in heat stress condition (*P* < 0.05; Fig. 1A). The progesterone was 2.04 fold greater in the CON cows compared with the HS cows (*P* < 0.05). The cortisol, prolactin and substance P, were 2.77, 1.40 and 1.67 folds, respectively, and the levels were greater in the HS cows compared with the CON cows (*P* < 0.05). Isoprostane and PGFM were 1.28 and 1.39 folds, respectively, greater in the HS cows compared with the CON group (*P* < 0.05).

#### Association of THI, hormone concentrations and number of transferrable embryos on gestational Day 7

Associations of THI and hormone concentrations on GD-7 and the number of transferrable embryos recovered on GD-7 were presented in Table 3. THI negatively, progesterone positively, and isoprostane-8 negatively were associated with the number of transferrable embryos recovered on GD-7 (*P* < 0.05; r^2^ = 0.43).

**Table 3:** Multiple regression analysis for the association of temperature humidity index and hormone concentrations with the number of transferrable embryos recovered on Day 7 in dairy cows.

Predictor	Estimate	Standard error	t statistic	*P* value	R^2^
Constant	3.01	1.42	–	–	0.43
THI§	− 2.95	0.55	4.39	0.001	
Progesterone	3.52	0.76	5.11	0.001	
Isoprostane-8	− 1.84	0.46	3.43	0.04	

^1^Refer Fig. 3b for protocol;

^§^Temperature humidity index.

Rectal temperatures (° C) in the HS and CON groups were 38.3 ± 1.2 and 38.0 ± 1.1 on Day 0, 38.5 ± 0.9 and 38.3 ± 1.2 on Day 9, and 38.4 ± 1.3 and 38.1 ± 1.0 on Day 16, respectively (*P* > 0.1).

### Experiment 2

Overall, the length, width, and weight of GD-16 conceptuses varied from 12 to 85 mm, from 1.03 to 5.05 mm and from 12.4 to 72.1 mg, respectively.

#### Morphometry of gestational Day 16 conceptuses

The mean (± SEM) length of embryo differed between cows under heat stress and control condition, 45.2 ± 10.6 mm and 59.2 ± 9.1 mm, respectively (*P* < 0.05). The mean (± SEM) width did not differ between cows under heat stress and in control condition, 3.70 ± 1.60 mm vs. 4.33 ± 1.21 mm, respectively (*P* > 0.1). Between cows in the HS and CON groups, mean (± SEM) weight (31.4 ± 4.3 vs. 42.4 ± 6.2 mg) differed (*P* < 0.05).

#### Conception rate and conceptus elongation on Day 16

The CR was greater for cows in the CON group compared with the HS group, 52 (52/100) vs. 31% (31/100), respectively (*P* < 0.01). The % filamentous conceptus recovered from cows in control condition was greater compared with cows in heat stress group, 71 (37/52) vs 45% (14/31), respectively (*P* < 0.05). The % tubular conceptus recovered from cows in the CON group was lower compared with cows in the HS group, 29 (15/52) vs 55% (17/31), respectively (*P* < 0.01).

#### Serum hormone concentrations on gestational Day 16

The mean (± SEM) serum progesterone concentration was greater and cortisol, prolactin, substance-P, Isoprostane-8 and PGFM levels were lesser in cows under control condition compared with cows in heat stress condition (*P* < 0.05; Fig. 1B). The progesterone was 2.09-fold greater in the CON cows compared with the HS cows (*P* < 0.05). The cortisol, prolactin and substance-P were 1.86, 1.60 and 1.55 folds, respectively, and the levels were greater in the HS cows compared with the CON cows (*P* < 0.05). Isoprostane and PGFM were 1.34 and 1.97 folds, respectively, greater in HS cows compared with CON cows (*P* < 0.05).

#### Association of THI, hormone concentrations, and conceptus length on gestational Day 16

Association of THI, hormone concentrations and conceptus length on GD-16 were presented in Table 4. The THI was negatively, progesterone was positively, and substance-P and isoprostane-8 were negatively associated with the length of GD-16 conceptus (*P* < 0.05; r^2^ = 0.52).

**Table 4:** Multiple regression analysis for the association of temperature humidity index and hormone concentrations with conceptus length on gestational Day 16^1^ in dairy cows.

Predictor	Estimate	Standard error	t statistic	*P* value	R^2^
Constant	2.98	1.87	–	–	0.52
THI	− 2.11	0.49	3.85	0.01	
Progesterone	3.13	0.61	4.99	0.001	
Substance-P	− 1.93	0.73	3.73	0.01	
Isoprostane-8	− 2.02	0.52	3.67	0.02	

^1^Refer Fig. 4 for protocol;

^§^Temperature humidity index.

Rectal temperatures (°C) in the HS and CON groups were 38.4 ± 1.1 and 38.1 ± 1.3 on Day-10, 38.2 ± 1.1 and 37.9 ± 0.9 on Day 0, and 38.5 ± 1.3 and 38.3 ± 1.0 on Day 7, respectively (*P* > 0.1).

## Discussion

In cattle, heat stress has deleterious effects on physiological functions including reproductive processes. Exposure of dairy cows to a high ambient temperature causes a decrease in the length and intensity of estrus by disrupting ovarian function as well as a decline in pregnancy rate following artificial insemination.

In the current study, heat stress resulted in lower CR, small conceptus and reduced conceptus weight in cows. the THI was negatively correlated with the GD-16 conceptus length, illustrating that optimal temperature and relative humidity are the most critical factors required for successful early embryonic development. Conceptus length during maternal recognition of pregnancy was found to be a signal of its developmental quality and the likelihood of influencing the establishment and maintenance of pregnancy^35^. Even though the vulnerability of the bovine embryo to heat stress after Day 7 is unclear, there is a claim that embryo survival is not dependent on the maternal heat stress beyond Day 7^36^. Nevertheless, Biggers et al. (1987) reported that heat stress from Day 8 to 16 reduced the conceptus weight at Day 16 in beef cattle^37^. Though the progesterone concentrations in heat stressed cows were comparable to control cows, the CL wet weight was reduced in heat-stressed cows in that study.

Some studies revealed that exposing cows to acute heat stress was not associated with a reduction in progesterone concentration^14,38,39^. The increased concentration of progesterone observed in those cases was associated with the adrenal secretion of progesterone in acute heat stress^38,39^. Interestingly, significant decrease in progesterone was typically observed when cows were exposed to chronic, seasonal heat stress^15^. This can be ascribed to disruption in the CL formation and function following abnormal development of preovulatory follicles in heat stressed cows. In the present study, decrease in progesterone concentration was noticed in heat-stressed cows and the decrease was associated with reduced transferable embryo yield on GD-7 and conceptus length on GD-16. It should be noted that Carter et al. (2008) observed an increase in progesterone concentration and a larger conceptus on GD 13 and 16 following supplementation of progesterone from GD 3 onwards substantiating the importance of progesterone concentrations^40^. Though increased cortisol was observed in heat-stressed cows in the current study, it was neither associated with the transferable embryo yield on GD-7 nor associated with the conceptus length on Day 16.

Subclinical endometrial inflammation caused reduced CL volume, lowered serum progesterone concentration and negatively affected GD-16 conceptus length in dairy cows^22^. In the current study, Isoprotane-8 was at higher concentration in heat-stressed cows and was negatively affected the transferrable embryo yield on GD-7 and conceptus length on GD-16. Although Trout et al. (1998) reported that heat stress did not increase lipid peroxidation or decrease lipid-soluble antioxidant concentrations in blood^39^, the effect of short-term acute heat stress was investigated in that study. Increased Isoprotane-8 levels in heat-stressed cows and cows with subclinical endometritis may be plausibly due to elevated ROS. Collectively, suboptimal CL function and adverse uterine environment due to ROS in heat-stressed cows could have resulted in reduced conceptus length.

In the current study, we investigated the impact of heat stress on conception and embryo quality following single insemination, and embryo yield following superovulation on GD-7. The CR and % transferable embryo yield were lesser in heat stressed cows on GD-7. The production of embryos by superovulation was negatively affected and embryonic development was compromised during the hot seasons^41^. De Rensis and Scaramuzzi (2003) postulated that heat stress may increase stress hormone levels and potentially impair early embryonic development^42^. It has been shown that heat stress increases oxidative markers levels such as TBARS, superoxide dismutase (SOD) and catalase, in plasma and erythrocytes in cows^43^. Heat stress increased intracellular reactive oxygen species (ROS) in bovine embryos and affected the development of early embryo^44^. On GD-7, heat stressed in vitro bovine embryos had increased reactive oxygen species and decreased IFNT expression in comparison to the control^45^. Yoon et al. (2013) demonstrated that excessive reactive oxygen species (ROS) reduced the embryo development rate and increased the number of apoptotic cells in embryos cultured *in vitro*^46^. Cows in the heat stressed group had increased unfertilized oocyte and degenerate embryo in the current elucidation, and the impact due to heat stress plausibly resulted in poor oocyte quality and poor development of early embryos.

In the current study, stress hormones cortisol, prolactin and substance-P were found at greater concentrations in the HS cows. Further, Isoprostane-8, a biomarker of oxidative stress, was also increased in the HS cows in this study. Increased level of stress hormones is associated with oxidative damage. Isoprostanes directly contributed to the functional consequences of oxidative stress (e.g., via activation of the prostanoid receptor) by affecting endothelial cell function and regeneration, vascular tone, hemostasis, and ischemia/reperfusion injury^47^. The intrauterine environment was compromised in heat stressed cows by decreasing blood flow to the uterus^48,49^. The progesterone concentrations on GD-7 in the HS cows were lower in the current study. The activity of antioxidants enzymes, such as CAT, GPx and SOD2, were up regulated in sheep endometrium as pregnancy progressed. Progesterone regulated GPx activity^50,51^ and glutathione reductase levels in rats^52^, as well as SOD1, CAT and GPx activities in sheep^53^. It should be noted that results from our lab showed that the isoprostane-8 and progesterone concentrations were negatively correlated, and progesterone concentrations were positively correlated to CL volume in cows under stress conditions^22,54^. Additionally, beef cows with small CL exhibited increased lipid peroxidation and reduced GPx and CAT enzyme activities. Collectively, heat stress inhibited embryonic development and increased early embryonic loss by negatively altering CL function and uterine environment^55^^.^^56^.

Additionally, PGFM concentrations were increased in both experiments in the current study. Elevated uterine luminal concentrations of PGF2a have been negatively associated with embryo quality and pregnancy rates^57^ and have been shown to have a toxic effect on in vitro development of embryos in cows^58,59^. It should be noted that the main plasma metabolite of PGF2a is 13,14-dihydro-15-keto-PGF (PGFM)^60^, and assay of PGFM has been used as an indicator of PGF release into the circulation^61^. In vitro studies showed that heat stress significantly increased phospholipase A2 (PLA2), cyclooxygenase 2 (COX2), prostaglandin F synthase (PGFS), prostaglandin E synthase (PGES), and carbonyl reductase 1 (CBR1) mRNA expression in the uterine stromal cells. This suggest that HS induces mRNA expression of enzymes involved in PG synthesis, resulting in the upregulation of PGE2 and PGF2α production in the stromal cells^62^. In pregnant rats, heat increased placental PGF2α and PGFM levels^63^. It should be noted that circadian rhythm mediates prolactin and glucocorticoids release^64^, and PGFM released^65^ in pulses in dairy cows. The readers need to be cautious when interpreting the results of hormones from the present study since they were measured once on respective days.

It should be noted that the THI denoting heat stress level has been variously categorized by different researchers, and disparities in definitions and classifications were overlooked between studies and conditions^66^. The consequences due to heat stress are severe in southwestern United States and Brazil where the summer season is long; however, animals in central Europe, northern United States and Canada are also affected by heat stress, where the summer season is relatively short but hot and there is a minimal decline in overnight temperatures^67^. It should be noted that poor reproductive performance during summer months has been described in intensively managed lactating cows with the air temperatures of 25 to 28 °C in cool regions of the world^4^. Armstrong et al. (1994) categorized THI of < 71 as normal, 72 to 79 as mild, 80 to 90 as moderate, and > 90 as severe heat stress^68^. Relatively, De Rensis et al. (2015) categorized THI < 68 as normal, THI of 68 to 74 as mild, and THI ≥ 75 as severe heat stress indicators^69^. Schüller et al. (2013) compared the AT, RH, and the resulting THI data obtained from seven different barns with those data obtained from the closest official meteorological stations. The authors found that the THI was higher (11.1 ± 6.5) in the barn than at the official meteorological station^28^. The current study utilized the calculated THI from the barn, and THI of ≥ 73 and of < 73 were used as cut-off for heat stress and control groups, correspondingly.

Nevertheless, heat stress during the period of breeding was consistently associated with reduced CR^2,70^. Furthermore, negative effects of heat stress have been noticed from 42 days before to 40 days after insemination^9^. Schüller et al. (2014) reported that the most negative impact of heat stress on CR was observed 21 to 1 day before breeding^10^. Cows in an environment with a mean THI of 73 or more were 61% less likely to get pregnant than those in a surrounding with a mean THI of < 73 during the period from Day 21 to 1 before breeding^10^. Hence, the onset of experiment in the heat stress group was started at least 3 weeks after the beginning of summer.

It should be noted that the current study was a field trial and cows included were not contemporary, therefore, other factors such as diets, management, and housing were not evaluated and could have impacted the results. Elevated rectal temperature and increased respiratory rate were noted as thermal stress indicators. The rectal temperatures of cows in HS and CON groups at initiation of synchronization or superovulation, and on the day of artificial insemination and embryo collection were within normal limits in the current study. It is plausible that the cows may have increased their water intake because of the heat stress as a mechanism to dissipate heat and may have led to normal rectal temperature when the temperatures were measured. Thus it is advisable to monitor rectal temperatures and respiratory rate constantly throughout the experimental period which is the limitation of the study. Burfeind et al., reported 7.4% and 28.1% of healthy dairy cows had elevated rectal temperature in the moderate (THI: 59.8 ± 3.8) and hot period (THI: 74.1 ± 4.4)^71^. This suggests that 72% of healthy cows have normal rectal temperature during heat stressed conditions. Kauffman et al. (2018) claimed vaginal temperature showed stronger relationships with THI compared with rectal temperature^72^. Li et al. (2020) proposed a model including ambient temperature, relative humidity (RH), wind speed (WS), milk yield (MY), time blocks, rectal temperature, and respiration rate and claimed that the model was better at suppressing prediction error and had better sensitivity and accuracy in recognizing thermal stress^73^. Although rectal temperature and respiratory rates were not constantly recorded in the current study, environmental THI was continuously monitored throughout the experimental period and the barn THI ≥ 73 was used as criteria to define HS and CON. It should be noted that mean THI ≥ 73 for an hour exposure per day decreased the CR significantly^10^.

As noted in previous studies, the early embryonic development up to GD-7 was reduced plausibly by compromised oocyte and suboptimal uterine environment. Further, the current study was the first to report on the impact of pre-breeding heat stress on the development of embryo beyond GD-7. In the current study, in addition to the decline of the % CR on GD-16, both conceptus length and weight were reduced in the heat-stressed cows.

**Figure 2:**
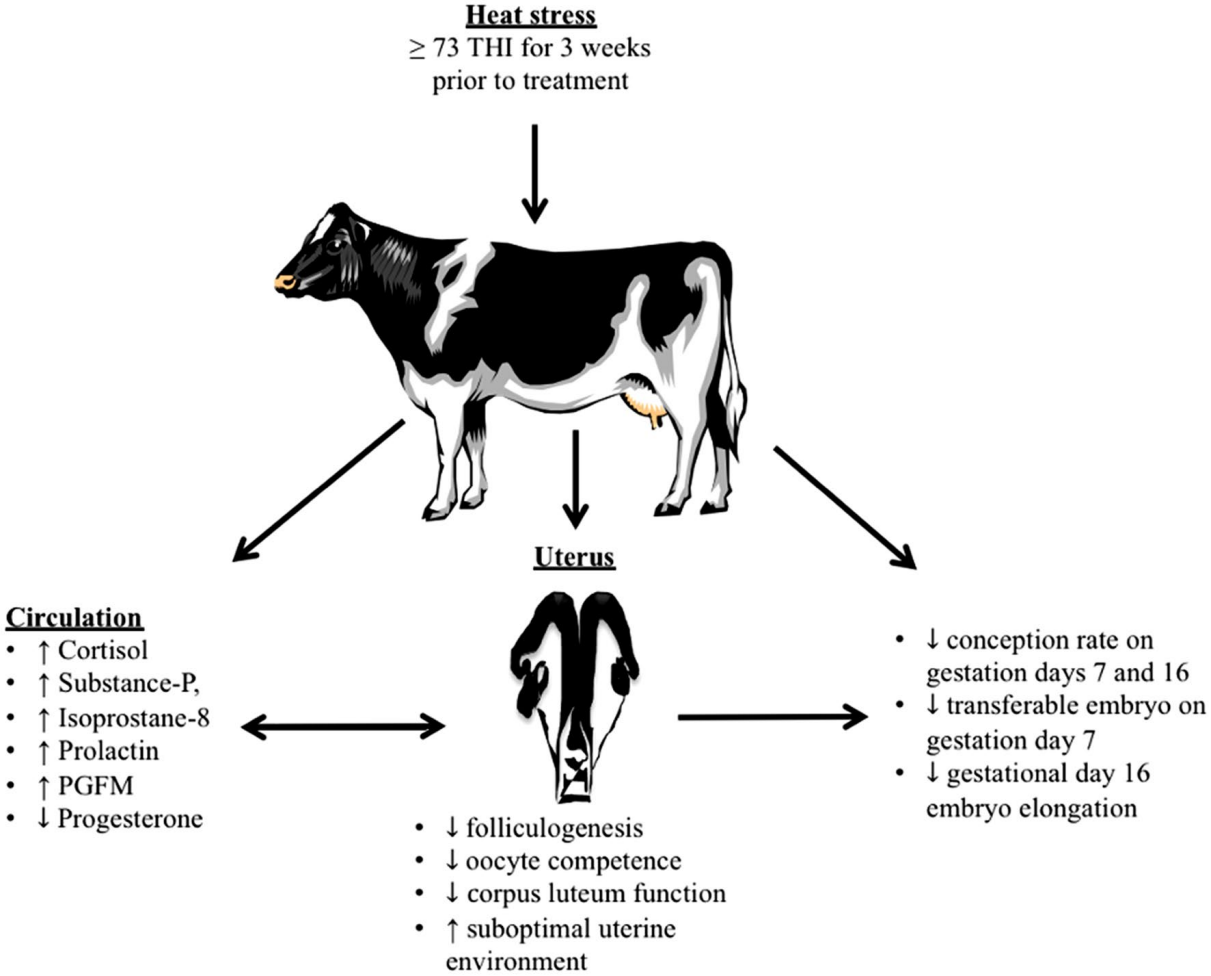
Effect of heat stress on reproductive function in dairy cows. Heat stress causes increase in stress hormones (cortisol, substance-P, and prolactin) resulting in poor folliculogenesis and oocyte competence; reduces corpus luteum function leading to decrease progesterone; and increases isoprostane-8 and PGFM resulting in suboptimal uterine environment. Together altered hormones and consequential disruption of luteal function and uterine environment resulted in decreased conception rate on gestational day 7 and 16 following single insemination, decreased transferable embryos on gestational day 7 following single insemination and superovulation and compromised elongation of gestational day 16 embryo. The figure was created using BioRender (https://www.biorender.com).

The pathophysiology of heat stress on reproductive function in dairy cows is presented in Fig. 2. Heat stress in dairy cows causes—1. Increased (a) cortisol, (b) substance-P (c) prolactin and (d) PGFM in circulation, leads to 2. (i) compromised (a) folliculogenesis, (b) oocyte competence and (c) corpus luteum function and resulting decreased progesterone, and (ii) increased (a) isoprostane-8 and (b) PGFM resulting suboptimal uterine environment, leads to 3. Decreased early embryo development—(a) decreased conception rate on GD-7 and GD-16, (b) decreased transferable embryos on GD-7 following superovulation and (c) compromised elongation of GD-16 embryo.

## Conclusions

Heat-stressed cows produced fewer Day 7 transferable embryos and had poorly elongated embryos and reduced conception rate on gestational Day 16. Increased stress hormones, isoprostane-8, and PGFM could have plausibly reduced the progesterone concentrations in heat-stressed cows. Further, increased isoprostane could have plausibly caused the suboptimal CL function and the adverse uterine environment in the heat-stressed cows, and consequently resulted in poor embryonic development during this period.

## Methods

This study was performed in compliance with appropriate ethics, standard operating procedures, handling and use of animals, sample collection and use of biomaterials for research (https://www.adsa.org/Portals/_default/SiteContent/docs/AgGuide3rd/Ag_Guide_3rd_ed.pdf). All procedures involving the use of animals were also conducted in accordance with the guidelines for agricultural animal care by the Washington State University (https://iacuc.wsu.edu/documents/2016/06/policy_7.pdf/).

The experiments were conducted on a commercial dairy farms in the pacific northwest region of the USA, from 2016 to 2019. Temperatures in the region can vary greatly with summertime highs in the upper 80s during the day and the lower 60s at night. The temperature in the wintertime varies with highs in the upper 40s and lows in the lower 30s. The barn was positioned in a southwest-northeast orientation with open ventilation and a mechanical fan system. Reproductive management included insemination following visual observation of estrus behavior, use of fixed time insemination protocols (Ovsynch)^21^, and PGF2a program in open cows following pregnancy diagnosis. In addition, a selective embryo transfer program for first or second service and selective natural service of cows beyond 200 DIM were also employed.

### Experiment 1

#### Experiment 1 a

##### Cows

Lactating Holstein Friesian cows (parity 2 to 4), without a history of peripartum metabolic disorders, dystocia, retained placenta, postpartum uterine diseases, mastitis, or lameness were selected in this study^22^. Besides, these cows were healthy and their body condition score (BCS: 1 emaciated, 5 obese) ranged from 2.5 to 3.5^23^. At the time of enrolment, cows were in between 60 and 90 days in milk (DIM) and were housed in free-stall barns with slatted floors and free-stalls equipped with rubber mats. Cows were not submitted to breeding before the enrolment. All cows were fed twice daily with a whole mixed ration (TMR) formulated to meet or exceed dietary needs for cows weighing ~ 520 to 710 kg and producing ~ 25 to 37 kg of 3.5% fat-corrected milk.

##### Synchronization treatment

**Figure 3:**
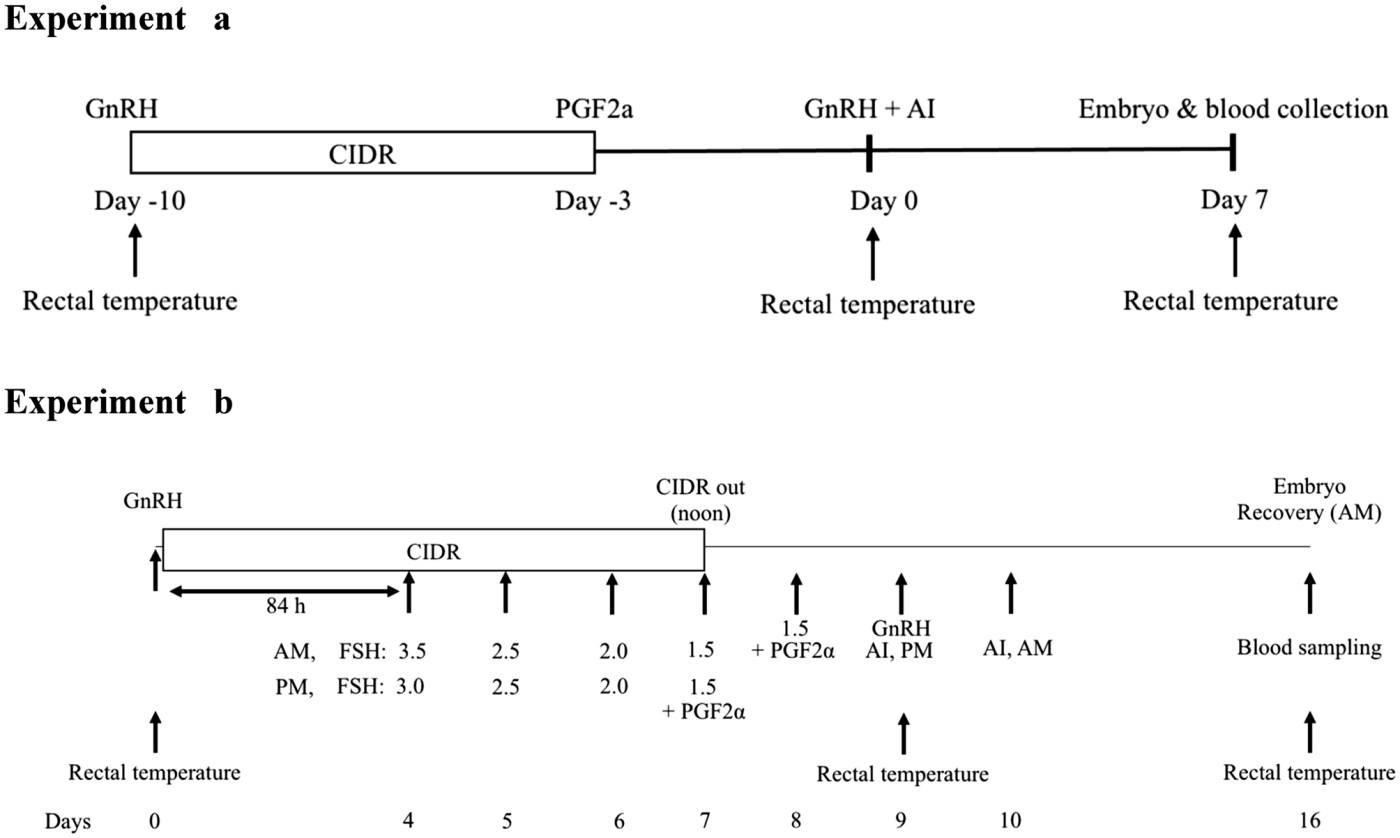
The schematic presentation of superovulation and embryo collection protocol. Experiment 1, a: All cows were fitted with a 1.3 g progesterone intravaginal insert (CIDR, Eazi-Breed CIDR Cattle Insert; Zoetis Animal Health, New York, NY, USA) and received 100 µg of gonadorelin hydrochloride (GnRH; 2 mL; im, Factrel; Zoetis Animal Health) on Day-10. On Day-3, CIDRs were removed, and 25 mg of dinoprost (PGF2α; 5 mL; im; Lutalyse sterile solution; Zoetis Animal Health) was administered to all cows. Cows in estrus were inseminated once 66 h after CIDR removal and administered with 100 µg of GnRH (im, Zoetis Animal Health) concomitantly (Day 0). Embryos were recovered by non-surgical uterine flush technique on Day 7. Rectal temperatures were recorded on Day-10, -3 and 7, and blood was collected on Day 7. Experiment 1, b: On Day 0, embryo donor cows received progesterone releasing vaginal insert (CIDR; 1.38 g of progesterone; Eazi-Breed CIDR Cattle Insert; Zoetis Animal Health, New York, NY, USA) along with gonadorelin hydrochloride (GnRH; 2 mL (100 µg), im, Factrel; Zoetis Animal Health). On Day 4 (84 h after GnRH), superovulation with Folltropin-V (Follicle stimulating hormone (FSH) equivalent to 400 mg NIH-FSH-P1; im; Bioniche Animal Health, Athens, GA, USA) was initiated, twice daily decreasing doses over 4.5 days. Luteolytic dose of dinoprost (PGF2α; 5 mL (25 mg) im; Lutalyse sterile solution; Zoetis Animal Health) was administered with the last two FSH injections and progesterone inserts were removed with the second last FSH injection, on Day 7 PM. Ovulation is induced with GnRH (100 µg im; Zoetis Animal Health) 44 h after progesterone insert removal and then donors are artificially inseminated fixed-time at 12 and 24 h later. Day 7 embryos were recovered by non-surgical uterine flush technique on Day 16. Rectal temperatures were recorded on Day 0, 9 and 16, and blood was collected on Day 16.

Cows in the control group (CON; n = 100) exposed to THI < 73, 3 weeks prior to initiation of synchronization protocol in early spring (Day-21), whereas cows in the heat stress group (HS; n = 100) experienced a THI of ≥ 73, 3 weeks prior to initiation of synchronization protocol in summer months (-Day 21)^10^. All cows were synchronized using a CO-Synch + CIDR protocol (Fig. 3a). Briefly, a 1.3 g progesterone intravaginal insert (CIDR, Eazi-Breed CIDR Cattle Insert; Zoetis Animal Health, New York, NY, USA) and a 100 µg of gonadorelin hydrochloride (GnRH; 2 mL; im, Factrel; Zoetis Animal Health) were administered on Day-10. The CIDRs were removed, and 25 mg of dinoprost (PGF2α; 5 mL; im; Lutalyse sterile solution; Zoetis Animal Health) was injected to all cows on Day-3. Cows were inseminated once 66 h after CIDR removal, and a 100 µg of GnRH (im, Zoetis Animal Health) was administered concomitantly (Day 0). The AI sires (n = 4) were randomly assigned to donor cows. The sire conception rate (SCR) score of the AI sires were ≥ + 4.

##### Embryo collection

Embryos were collected non-surgically from donor cows on Day 7 after AI. A sterile 16 Fr. two-way Foley catheter with a 5 cm^3^ balloon (Agtech Inc., Manhattan, KS, USA) was positioned in the uterine body and embryo collection medium (Agtech Inc.) was introduced by gravity flow through “Y”-junction tubing. The embryo was flushed individually with approximately 250 to 300 mL of medium and the medium was then passed through an Em-Con Embryo Collection Filter (Agtech Inc.). A small volume of recovered fluid was retained on the filter. The recovered fluid was shifted from the embryo filter into a flat, gridded search petri dish and viewed under a stereoscope for the presence of embryo. Once found, the embryo was transferred to another petri dish containing holding medium and were evaluated for their quality and stage of development as described in the international embryo transfer society (IETS) guidelines for classification of bovine embryos^24^.

#### Experiment 1 b

##### Cows

Upon applying similar screening and selection criteria as in the experiment 1a, Lactating Holstein Friesian cows (n = 20, HS = 10 and CON = 10; parity 2 to 4; BCS 2.5 to 3.5) were selected. At the time of enrolment, cows were in between 60 and 90 days in milk, housed in free-stall barns and were not submitted to breeding before the enrolment. All cows were fed twice daily with a TMR prepared to meet or exceed dietary needs for cows weighing ~ 520 to 690 kg and producing ~ 25 to 37 kg of 3.5% fat-corrected milk.

##### Superovulation treatment

The schematic presentation of superovulation and embryo collection protocol was given in Fig. 3b. Ten cows (CON group) underwent SO treatment in early spring (THI < 73 for 3 weeks from Day-21)^10^. Ten cows (HS group) underwent SO treatment in summer months (THI ≥ 73 for 3 weeks from Day-21)^10^. In Brief, on Day 0, cows’ reproductive tract was palpated per-rectally, and scanned transrectally using ultrasonography (Aloka 500, with 5 MHz linear transducer, Sysmed Lab, Inc., Chicago, IL, USA). Cows with a CL > 1.5 cm in size and with normal reproductive tract were selected. All chosen embryo donor cows were fitted with progesterone releasing vaginal insert (Zoetis Animal Health) and were administered with an injection of 100 µg of GnRH (im, Zoetis Animal Health). On Day 4 (84 h after GnRH injection), superovulation using Folltropin-V (Follicle stimulating hormone (FSH) equivalent to 400 mg NIH-FSH-P1; im; Bioniche Animal Health, Athens, GA, USA) was begun with twice daily administration of a decreasing dose over 4.5 days. Dinoprost tromethamine injections (25 mg, im Zoetis Animal Health) were simultaneously administered with the last two FSH injections, and progesterone inserts were removed with the second to the last FSH injection, on Day 7 PM. Ovulation was induced by 100 µg of GnRH (im; Zoetis Animal Health) injection, 44 h after removal of progesterone insert. The donors were artificially inseminated, with frozen-thawed semen at a fixed-time, 12 and 24 h after GnRH injection. The sires (n = 2) were randomly allocated to donor cows. The SCR score of the AI sires were + 4.

##### Embryo collection

Embryos were collected non-surgically from the donor cows on Day 7 after second AI. A sterile 16 Fr. two-way Foley catheter with a 5 cm^3^ balloon (Agtech Inc., Manhattan, KS, USA) was positioned in the uterine horn and embryo collection medium (Agtech Inc.) was infused by gravity flow through “Y”-junction tubing. Each uterine horn was flushed individually with approximately 200 to 250 mL of medium (3 to 5 flushes per horn) and the medium was passed through an Em-Con Embryo Collection Filter (Agtech Inc.). A small volume of fluid was retained on the filter. The recovered fluid was transferred from the embryo filter into flat, gridded search petri dishes and viewed under a stereoscope for presence of embryo. Once identified, the embryos were transferred to another petri dish containing holding medium and were evaluated for their quality and stage of development as described in the international embryo transfer society (IETS) guidelines for classification of bovine embryos^24^. Zero response was defined as cows with no CL and zero embryo yield following superovulation treatment.

### Experiment 2

#### Cows

Lactating Holstein cows (n = 200, parity 2 to 4) were selected after employing similar screening and selection criteria as in the experiment 1. At the time of enrolment, cows were in between 60 and 100 DIMs, housed in free-stall barns and were not submitted to breeding prior to the enrolment. All cows were fed twice daily with a TMR formulated to meet or exceed dietary needs for cows weighing ~ 520 to 710 kg and making ~ 25 to 37 kg of 3.5% fat-corrected milk.

#### Synchronization

**Figure 4:**
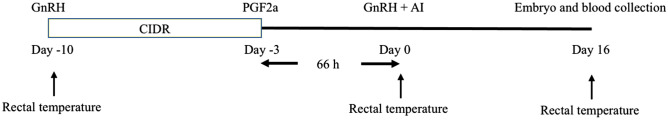
The schematic presentation of CIDR + CO-Synch protocol. All cows were fitted with a 1.3 g progesterone intravaginal insert (CIDR, Eazi-Breed CIDR Cattle Insert; Zoetis Animal Health, New York, NY, USA) and received 100 µg of gonadorelin hydrochloride (GnRH; 2 mL; im, Factrel; Zoetis Animal Health) on Day-10. On Day-3, CIDRs were removed, and 25 mg of dinoprost (PGF2α; 5 mL; im; Lutalyse sterile solution; Zoetis Animal Health) was administered to all cows. Cows in estrus were inseminated once 66 h after CIDR removal and administered with 100 µg of GnRH (im, Zoetis Animal Health) concomitantly (Day 0). Embryos were recovered by non-surgical uterine flush technique on Day 16. Rectal temperatures were recorded on Day-10, -3 and 7, and blood was collected on Day 16.

Cows in the CON group (n = 100) were experienced THI of < 73 in early spring for 3 weeks prior to initiation of synchronization protocol, whereas cows in the HS group (n = 100) exposed to a THI of ≥ 73 in summer months for 3 weeks prior to initiation of synchronization protocol (-Day 21)^10^. A CO-Synch + CIDR protocol (Fig. 4) was used to synchronize all cows, similar to the experiment 1a. The AI sires (n = 4) were randomly allocated to donor cows. The SCR score of the AI sires were ≥ + 4.

#### Embryo collection

Day 16 conceptuses were collected from all cows. Briefly, conceptuses were collected on the Day 16 by standard non-surgical uterine flushing technique using an 18-g embryo collection catheter (AgTech Inc., Manhattan, KS, USA) in Phosphate Buffered Saline (PBS; pH 7.4)^22,25^. Conceptuses were then washed in PBS and viewed under naked eye and under a stereomicroscope. The width and length were measured, and weight was calculated (total wet weight of embryonic disc and trophoblast was measured after placing in the blotting paper for 1 min. Conceptus weight was computed by deducting the weight of blotting paper from the total weight). Care was taken while flushing uterus to ensure recovery of intact conceptuses, especially by controlling the flow of flush medium entering the uterus and flow of recovery. Completely fragmented conceptuses were excluded from the study. Based on the length, conceptuses were categorized as tubular (10 to 20 mm) or filamentous (≥ 25 mm) embryo^26,27^.

#### Temperature humidity index

In all experiments, ambient temperature (AT, °C) and relative humidity (RH, %) were recorded using an automated data logger (Tinytag Plus 2, Micron Meters, Tucker, Georgia, USA) in the barns^28,29^ during the course of the experimental period. Daily THI was calculated using the equation reported by Kendall and Webster (2009)^30^: THI = (1.8 × AT + 32) − [(0.55 − 0.0055 × RH) × (1.8 × AT − 26)]. Further, rectal temperature was recorded once on Days-10, 0, and 7 in experiments 1a and 2 and on Days 0, 9, and 16 in experiment 1b using GLA M900 Livestock Thermometer (Tech Instrumentation Inc., Elizabeth, CO, USA).

##### Blood collection and hormones analysis in Experiments 1 and 2

Blood samples were collected by coccygeal venipuncture on the day of embryo collection in experiment 1b (Day 7 after AI), and in experiment 2 (Day 16 after AI) into vacutainer tubes (Becton Dickinson, Franklin Lakes, NJ, USA). The blood samples were left to clot at room temperature for 15 min and stored at 4 °C. The samples were centrifuged at 1500×*g* for 20 min at 4 °C within an hour of collection and serum was separated and stored at − 20 °C until further utilization. Separate blood samples were collected into vacutainer tubes containing EDTA (Becton Dickinson) to measure PGFM. The blood was centrifuged at 2600×*g* for 30 min, and the plasma was decanted and frozen at − 20 °C for analysis of the PGFM.

#### Cortisol and progesterone

Serum cortisol and progesterone concentrations (Enzo Life Sciences, Farmingdale, NY, USA) were determined by the method described previously^31–33^. Assay sensitivity for cortisol was 0.2 µg/dL and for progesterone was 0.02 ng/mL. The intra- and inter-assay coefficients of variation (CV) were 5.2% and 3.7% for cortisol and were 8.8% and 6.1%, for progesterone correspondingly.

#### Substance-P

Substance-P was extracted from the plasma samples as described previously^55,56^, stored at − 20 °C and subsequently reconstituted in assay buffer immediately before analysis. Average extraction efficiency was 80%. Substance-P ELISA kits (Enzo life Sciences, Farmingdale, NY, USA) were used according to the manufacturer’s instructions. Sensitivity of the assay was 0.008 ng/mL. The intra- and inter-assay CV were 6.7% and 4.3%, correspondingly.

#### Prolactin assay

Serum prolactin concentration was determined by competitive enzyme immunoassay technique following manufacturer’s recommendation using bovine prolactin ELISA kit (MBS721395, MyBioSource, LLC, San Diego, CA, USA)^32,33^. The intra- and inter-assay CV were 7.3 and 4.4%, correspondingly. Sensitivity of the assay was 0.01 ng/mL.

#### Isoprostane 8-epi-PGF2a

Isoprostane concentrations in serum samples were estimated employing direct ELISA^34^. Briefly, 100 μL of anti-goat-8-epi-PGF2a antibody (MyBioSource, LLC, San Diego, CA, USA) was added in their respective 96-well plates, that were pre-coated with differing concentration of standards or samples and incubated at 4 °C for at least 24 h. After washing with buffer, 100 μL of secondary antibody, raised in donkey anti-goat IgG-HRP (Santa Cruz Biotechnology, Inc.) was added to each well. After washing with buffer, 200 μL of reagent containing the substrate of acetyl cholinesterase was added, the contents were allowed to react, and then 50 μL of stop solution were added to pause the reaction. Plates were read at 450 nm and serum concentrations of isoprostane calculated from standard curves. The intra- and inter-assay coefficients of variation were 7.4 and 10.8%, correspondingly.

#### Prostaglandin F metabolites

Plasma PGFM concentrations were determined using an ELISA kit (Cayman Chemical, Ann Arbor, MI, USA)^32,33^. The manufacturer’s instruction was followed. Inter- and intra-assay coefficients of variation for one reference sample were 6.1 and 7.0%, respectively. Sensitivity of the assay was 0.02 ng/mL.

### Statistical analysis

Data analysis was performed using a statistical software (SAS Version 9.4, Cary, NC, USA) and *P* values ≤ 0.05 were considered statistically significant.

#### Experiment 1

Datasets were tested for normality distribution by Komogorov-Smirnov test and were then log 10 or arcsine transformed in case of missense. Cow was used as the experimental unit for embryo number and quality. The effect of heat stress on number of corpora lutea, total ova and embryos, transferable embryos, and embryo stages was tested for significance by PROC ANOVA and multiple comparisons using Tukey test.

#### Experiment 2

The CR (%) was calculated as number of cows yielded embryo on Day 16 divided by total number of cows inseminated. The differences in CR, % filamentous and tubular embryo yield on GD-16 between cows in the HS and CON groups were determined by PROC GLM. The difference in mean length, weight and width, and mean serum hormone concentrations on Day 16 between cows in the HS and CON groups were determined by PROC ANOVA with Duncan’s multiple range test. All data were evaluated for normality of their distribution using PROC Univariate method. Values were transformed using logarithmic, arcsine or square root transformations, but non-transformed data were presented. Multivariate regression analysis was performed using PROC REG method to determine the association of hormone concentrations and conceptus length.

Differences in rectal temperature between HS and CON groups were tested for normality using Shapiro–Wilk test. Transformed data (log10 or arcsine) were analyzed by one way ANOVA, with non-transformed values reported.

## Data Availability

The data that support the findings of this study are available from the corresponding author upon reasonable request.
